# Irinotecan-encapsulated double-reverse thermosensitive nanocarrier system for rectal administration

**DOI:** 10.1080/10717544.2016.1272651

**Published:** 2017-02-09

**Authors:** Fakhar ud Din, Ju Yeon Choi, Dong Wuk Kim, Omer Mustapha, Dong Shik Kim, Raj Kumar Thapa, Sae Kwang Ku, Yu Seok Youn, Kyung Taek Oh, Chul Soon Yong, Jong Oh Kim, Han-Gon Choi

**Affiliations:** 1 College of Pharmacy & Institute of Pharmaceutical Science and Technology, Hanyang University, Ansan, South Korea,; 2 Department of Pharmacy, Quaid-I-Azam University, Islamabad, Pakistan,; 3 College of Pharmacy, Yeungnam University, Gyongsan, South Korea,; 4 International Center of Chemical and Biological Sciences, University of Karachi, Karachi, Pakistan,; 5 College of Oriental Medicine, Daegu Haany University, Gyongsan, South Korea,; 6 School of Pharmacy, Sungkyunkwan University, Suwon, South Korea, and; 7 College of Pharmacy, Chung-Ang University, Seoul, Republic of Korea

**Keywords:** Irinotecan, double reverse thermosensitive nanocarrier, rectal administration, burst effect, toxicity, anti-tumor efficacy

## Abstract

Intravenously administered for the treatment of rectum cancer, irinotecan produces severe side effects due to very high plasma concentrations. A novel irinotecan-encapsulated double reverse thermosensitive nanocarrier system (DRTN) for rectal administration was developed as an alternative. The DRTN was fabricated by dispersing the thermosensitive irinotecan-encapsulated solid lipid nanoparticles (SLN) in the thermosensitive poloxamer solution. Its gel properties, pharmacokinetics, morphology, anticancer activity and immunohistopathology were assessed after its rectal administration to rats and tumor-bearing mice. In the DRTN, the solid form of the SLN and the liquid form of the poloxamer solution persisted at 25 °C; the former melted to liquid, and the latter altered to gel at 36.5 °C. The DRTN was easily administered to the anus, gelling rapidly and strongly after rectal administration. Compared to the conventional hydrogel and intravenously administered solution, it retarded dissolution and initial plasma concentration. The DRTN gave sustained release and nearly constant plasma concentrations of irinotecan at 1–3 h in rats, resulting in improved anticancer activity. It induced no damage to the rat rectum and no body weight loss in tumor-bearing mice. Thus, this irinotecan-encapsulated DRTN associated with a reduced burst effect, lack of toxicity and excellent antitumor efficacy would be strongly recommended as a rectal pharmaceutical product alternative to commercial intravenous injection in the treatment of rectum and colon cancer.

## Introduction

Irinotecan, a water-soluble anticancer drug, has been widely used in the therapy of metastatic carcinomas of rectum and colon as an intravenous administration (irinotecan hydrochloride injection™; West-ward Pharm. Co., Eatontown, NJ) (Kim et al., 2013; Sadahiro et al., 2015; Tran et al., 2015; Poudel et al., 2016). However, like other anticancer drugs, this drug produces severe cytotoxic side effects, such as body weight loss, irritation and diarrhea, owing to very high plasma concentrations. Thus, the practical study on its alternative pharmaceutical product with minimal side effects and excellent anticancer activity is necessary.

The conventional thermosensitive hydrogel system was developed by controlling the gelation temperature of a base, poloxamer solution which existed as a liquid at room temperature, but a gel in physiological temperature (Yong et al., 2004; Seo et al., 2013; Yeo et al., 2013). This hydrogel for rectal administration could be easily administered to the anus without outer leakage (Choi et al., 1998a, 1999). In addition, it could improve the bioavailability of the drug in animals and human subjects (Choi et al., 1998b; Yun et al., 1999; Yong et al., 2001; Seo et al., 2013). However, if this conventional hydrogel system is applied to a toxic drug, irinotecan, it may induce a very high initial dissolution of drug, resulting in high initial plasma concentration and *C*_max_ (maximum plasma concentration), followed by severe side effects similar to those observed following the intravenous administration.

In this study, to solve this demerit, a novel irinotecan-encapsulated double reverse thermosensitive nanocarrier system (DRTN) for rectal administration with reduced burst effect, minimal toxicity and excellent antitumor efficacy was developed. The DRTN was a dispersed hydrogel system in which the thermosensitive irinotecan-encapsulated solid lipid nanoparticles (SLN) were homogenously suspended within the thermosensitive poloxamer solution. In this DRTN system, the solid form of the former and liquid form of the latter persisted at 25 °C; however, the former melted to liquid and the latter altered to gel form at 36.5 °C (Din et al., 2015a). Its gel properties and dissolution were investigated. Additionally, compared to the conventional hydrogel and intravenously administered solution, its pharmacokinetics, morphology, anticancer activity and immunohistopathology were assessed after its rectal administration to rats and in tumor xenograft athymic nude mice. The SLN is a nanoparticlulate drug carrier for controlled drug delivery systems, because it gives the excellent release control and superior encapsulation of the drug (Ghadiri et al., 2012; Negi et al., 2013; Ramasamy et al., 2015).

## Material and methods

### Materials

Irinotecan (more than 99.0% purity) was obtained from Knowshine Pharma Chemicals Inc. (Shanghai, China). Poloxamer 407 (P407) and poloxamer 188 (P188) were purchased from BASF (Ludwigshafen, Germany). Span 20 was purchased from Sigma Aldrich (Steinheim, Germany). Tween 80 (polysorbate 80) was bought from DC Chem. (Seoul, South Korea). Triethanolamine and tricaprin were bought from Tokyo Chem. (Tokyo, Japan). All chemical reagents were used without further purification.

### Animals

Sprague-Dawley male rats (270 ± 20 g, 6–8 weeks old) for pharmacokinetic and rectum morphology study, and female athymic nude mice (20 ± 1 g, 5 weeks old) for anti-tumor and histopathology test were purchased from Nara Biotech. Co. (Seoul, South Korea). These animals were placed with free access to drinking water under the temperature of 21–22 °C and relative moisture of 55 ± 2% for acclimatization. The procedures for the animal studies were consistent with NIH Policy and the Animal Welfare Act under the approval of the Institutional Animal Care and Use Committee (IACUC) at Hanyang University.

### Irinotecan-encapsulated thermosensitive SLN

#### Fabrication

Irinotecan (1.150 g), 562.5 mg lipid mixture of tricaprin and triethanolamine (8:2, w/w), 112.5 mg Span 80, 450 mg Tween 80 and 7.725 g distilled water were sequentially mixed at 45 °C with gentle stirring. This solution was gradually cooled to room temperature after homogenizing at 45 °C utilizing an IKA homogenizer (T-8-Ultra-Turrax; Königswinter, Germany) and an Avestin high-pressure homogenizer (Emulsiflex B15; Ottawa, ON, Canada), leading to an irinotecan-encapsulated thermosensitive SLN dispersion.

#### Entrapment efficacy and drug content

One milliliter of the iriontecan-encapsulated SLN dispersion was diluted fourfold with normal saline and then centrifuged at 20 000 *g* for 2 h at 4 °C (Eppendorf 5430 R, Hamburg, Germany). The resulting solution (20 μl) was injected into the column Capcell Pak C18 MGS5 (Shiseido, Tokyo, Japan; 4.6 mm I.D. × 150 mm, 5 μm) at 40 °C to obtain the amount of non-encapsulated drug in the SLN. An Agilent-1260 HPLC system (Santa Clara, CA) was employed for the analysis. A mixture of sodium dibasic phosphate (25 mM, pH 3.1) and acetonitrile at 50:50 volume ratios was used as the mobile phase. For accurate drug assay, the eluent was monitored at 254 nm with a flow rate of 1 ml/min (Mathijssen et al., 2001; Xuan et al., 2006). Subsequently, entrapment efficacy was calculated as specified: entrapment efficiency (EE %) = (*W*_t_ − *W*_n_)/*W*_t_ * 100; “*W*_t_” represents total drug weight; “*W*_n_” indicates the weight of non-encapsulated drug in the thermosensitive SLN formulation. The equation used for determining the drug content was: Drug content (%) = *C*_p_/*C*_t_ * 100; *C*_p_ and *C*_t_ represent the practical and theoretical drug quantities, correspondingly.

### Double reverse thermosensitive nanocarrier system (DRTN)

#### Fabrication

The poloxamer solution was prepared by dissolving 15 g P407 and 17 g P188 in 54 g distilled water at 4 °C. This solution was kept in a refrigerator for 12 h to obtain a clear formulation. Ten grams of irinotecan-encapsulated SLN dispersion were sequentially introduced to this poloxamer solution with mild stirring at 4 °C, resulting in the formation of irinotecan-encapsulated DRTNs.

#### Particle size

The particle size of the irinotecan-encapsulated SLN in the DRTN was analyzed utilizing dynamic light scattering (Zetasizer Nano ZS; Worcestershire, UK) installed with a helium-neon laser and software (version 6.34). The SLN dispersion (10 μl) was distributed in 1 ml of deionized water and sonicated for 1 min. The analysis of its particle size was performed at a wavelength of 635 nm with a 90° fixed scattering angle in 25 °C. Each result displayed was measured three times.

### Transmission electron microscopy (TEM)

Hitachi H-7600 (TEM; Tokyo, Japan) was used to determine the morphology of the DRTN operating at 100 kV. In brief, DRTN samples were positioned on copper-grid (carbon-applied) and permitted to stick to the carbon plate. A droplet of 2% phosphotungstic acid solution was applied as negative staining.

### Gel properties

The glass vials each containing 4 g DRTN were taken with a magnetic bar (10 × 3 mm) inside for stirring purpose. An IKA digital thermometer (ETS-D5; Königswinter, Germany) was immersed into the glass vial. The temperature of DRTN was gradually amplified (1 °C/min), and the incessant speed of the magnetic stirring was 50 rpm. The temperature was elevated from 20 to 40 °C. The momentary temperature at which the magnetic bar stopped rotation was noted as the gelation temperature, a temperature at which the liquid phase converts into a gel state. Moreover, employing Brookfield viscometer (LVDV-II + P; Middleborough, MA) equipped with a software (RHEOCALC; Lorch, Germany), its gelation behavior was investigated at 36.5 °C, and its gel properties including gel strength and gelation time were also determined.

## Dissolution

Each DRTN and hydrogel containing 1.15% irinotecan (20 mg) taken in a gauze covered basket was placed in USP dissolution apparatus (Vision Classic 6, Hanson Research Co., Los Angeles, CA) containing 500 ml of distilled water as the dissolution medium. The dissolution test was performed at 36.5 °C and 100 rpm. At predetermined times, 1 ml of the media were sampled, filtered and assessed by the HPLC (high performance liquid chromatography) method as stated earlier**.**

### *In vivo* evaluation in rats

#### Pharmacokinetics

The pharmacokinetic study was carried out using three rat groups, each comprising six rats. They were placed on the operating board in supine condition after sedation with diethylether in a container. The right femoral arteries of the rats were cannulated for blood sample withdrawal. One group was intravenously injected with irinotecan solution at the drug dose of 25 mg/kg through the left femoral vein, while two other groups received DRTN and hydrogel at the same drug dose through the rectal route (4 cm in the rectum through a sonde fitted on a syringe), respectively. In this study, the intravascularly administered irinotecan was used as a control in order to determine the absolute bioavailability of irinotecan. At predetermined time intervals, the blood sample (300 μl) was withdrawn from the right femoral artery of each rat, followed by centrifugation at 9000 *g* for 10 min using Smart 15 microcentrifuge (Incheon, South Korea). Blood plasma obtained was stored in −20 °C for further examination. Each blood plasma (150 μl) was assorted with 150 μl acetonitrile and 10 μl camptothecin in acetonitrile solution (100 μg/ml) as the internal standard. Its centrifugation at 13 000 *g* was performed for 10 min to separate the proteins. Next, the supernatant (20 μl) was introduced into the column and assessed by the HPLC method as mentioned earlier**.**

#### Morphology

Hematoxylin and eosin (H&E) staining was carried out to investigate the morphological change in the rat rectum. After pharmacokinetic study, all rat rectums treated with DRTN and hydrogel were entirely removed. They were prepared for conventional sample treatment followed by H&E staining and investigated using an E400 microscope (Nikkon, Tokyo, Japan). The rectal epithelium with no treatment was used as the control. Investigation of deteriorating eruptions was performed, containing superficial epithelial shedding, inflamed cell penetrations and mucosal fibrotic variations. To detect the additional variations, mucosal and epithelial widths and statistics of mono nuclear cells were observed in rectal mucosa, and calculated with a computerized analysis using (*i*Solution FL ver 9.1; IMT *i*-solution Inc., Quebec, Canada).

### *In vivo* evaluation in tumor xenograft athymic nude mice

#### Anti-tumor efficacy

A tumor xenograft model was developed by subcutaneous injection of 1 × 10^6^ SCC7 (squamous cell carcinoma) cells suspended in cell culture media (100 μl) into the right flanks (thighs). The cell injection time was nominated as day 0, and the management of nude mice was initiated right after the tumor volume reached 100–150 mm^3^ (day 5) followed by formulation injection at day 8 and 11. The mice were distributed into three experimental (solution, hydrogel and DRTN treated) and a control group, with six mice in each group. DRTN and hydrogel were rectally administered to the respective groups at the drug dose of 5 mg/kg, while irinotecan solution was intravenously administered through tail vein injection at the same drug dose to the other group of mice. Untreated group of mice served as control. Calipers were used to measure the length and width of the tumor in individual mouse. Tumor volumes were calculated using the formula: *V* =  (*L* × W^2^)/2, where *V, L* and *W* represent volume, length and width, respectively. Variations in body mass were investigated to assess the harmfulness of respective test materials.

#### Histopathology and immunohistochemistry

The immuno-reactivity variations in the tumor of animals sacrificed after evaluation of anti-tumor efficacy were perceived in contradiction of the apoptotic markers, caspase-3 and PARP (cleaved poly(ADP-ribose) polymerase), angiogenesis marker, PECAM-1 (platelet endothelial cell adhesion molecule 1) or CD31 and tumor cell proliferation marker, Ki-67 (Ramasamy et al., 2015; Tran et al., 2015; Poudel et al., 2016). All the antibodies were used at a dilution of 1:500. Primarily, cleansed antibodies with Vectastain Elite ABC (avidin biotin-peroxidase complex) kit and peroxidase substrate kit were used for this purpose. Concisely, endogenous peroxidase was cultured in H_2_O_2_ (0.3%) and methanol for 0.5 h to block its activity. Similarly, horse serum blocking solution (Vector Labs; Burlingame, CA) was used to block nonspecific binding of immunoglobulin for 1 h at 90–100 °C and staining dish containing 10 mM citrate buffers of pH 6. Primary antiserum was preserved at 4 °C for 12 h followed by incubation at 25 °C and dilution of biotinylated universal secondary antibody (Vector Lab., Burlingame, CA. Dilution 1:50) and ABC reagents. Lastly, all the units were treated with peroxidase substrate kit at 25 °C for 3 min. After each phase, entire units were triply washed with 0.01 M PBS. The cells exhibiting higher (above 20%) immune-reactivity in the cytoplasm; the density, in contradiction of every antiserum (caspase-3 and PARP, CD31 and Ki-67) was observed as positive. The areas filled by these antiserum-positive cells situated in the tumor were calculated using automatic image analyzer (Park et al., 2014; Choi et al., 2015). Multiple comparison tests for statistical analysis were conducted. Variance homogeneity was analyzed employing the Levene test. If the Levene test gave no significant deviations, the data were checked using one way ANOVA test followed by least-significant differences (LSD) multi-comparison test. If the Levene test did, Kruskal–Wallis *H* test, a non-parametric comparison test, was performed. When a significant difference was observed in this test, the Mann–Whitney *U* test was used. Statistical analyzes were carried out employing SPSS for Windows (Release 14.0K, SPSS Inc., Chicago, IL). Differences were considered as significance at *p* < 0.05.

## Results and discussion

### Physicochemical characterization

**Figure 1.**
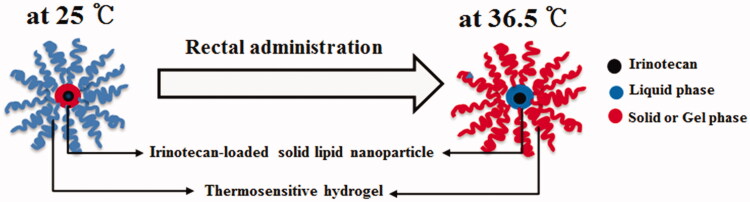
Schematic representation of double-reverse thermosensitive nanocarrier system.

The DRTN was manufactured with drug-encapsulated SLN and poloxamer solution, such that the former, were homogenously dispersed in the latter (Din et al., 2015a). It was destined that the solid form of the former and liquid form of the latter persisted at room temperature; on the contrary, the former melted to liquid and the latter altered to gel form in the body (Figure 1). The thermosensitive SLN dispersion consisted of irinotecan/lipid mixture/surfactant/water (1.15:0.5625:0.5625:77.25, weight ratio) showed high entrapment efficiency (almost 90%). The lipid blend of tricaprin and triethanolamine (8:2, weight ratio) with the melting point of approximately 32 °C is solid at 25 °C and melt at 36.5 °C (Din et al., 2015b). Moreover, the mixture of Tween 80 and Span 20 at the weight ratio of 4:1 was used as surfactant. Findings from our preliminary study presented that the added drug and surfactant scarcely influenced the melting point of lipid mixture in the SLN.

The DRTN comprised of 10 g SLN dispersion, 15 g P407, 17 g P188, 4 g Tween 80 and 54 g water that remained in the state of aqueous dispersion with the gelation temperature of about 32.5 °C, which was determined as the temperature at which the liquid change to gel (Yong et al., 2004; Yeo et al., 2013). Thus, this DRTN flowed easily at 25 °C so that it could be easily administered to the rectum by the sonde attached to the syringe (Yun et al., 1999; Yong et al., 2001; Seo et al., 2013). Conversely, after rectal administration, it was changed to gel form at the physiological temperature leading to direct opposition to the thermosensitive SLNs presenting the double reverse thermosensitive property (Figure 1).

**Figure 2.**
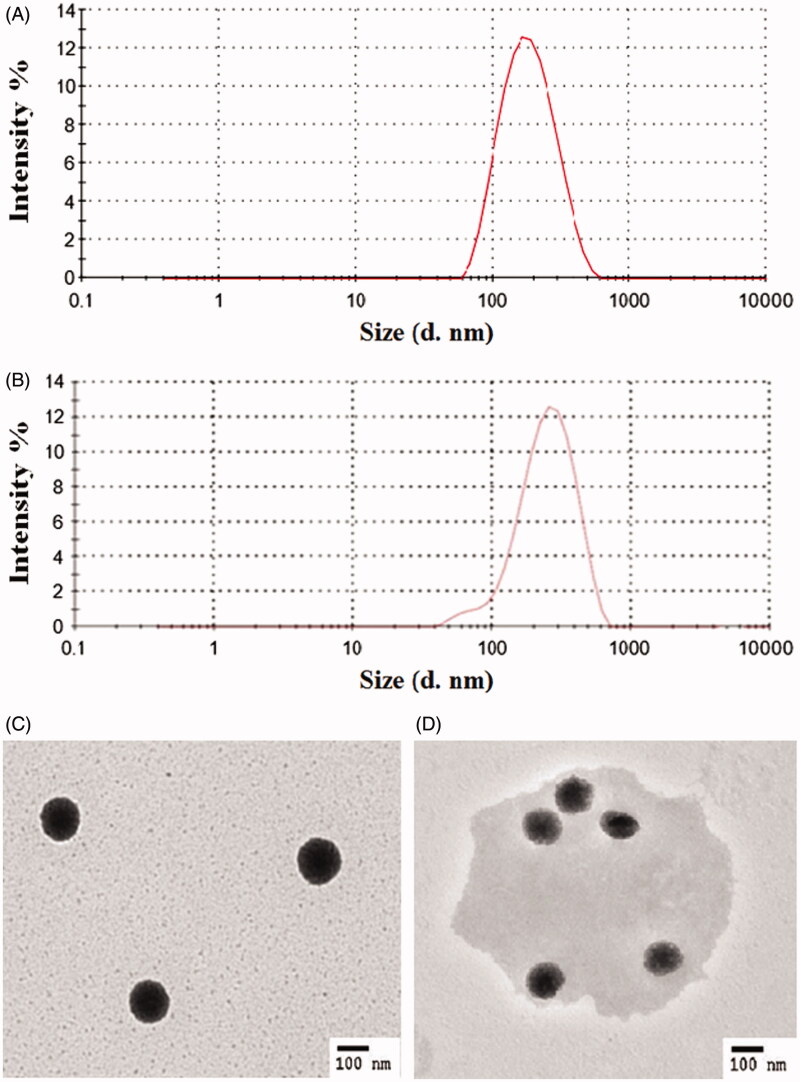
Particle characterization of SLN in the DRTN: (A) dynamic light scattering at 25 °C, (B) dynamic light scattering at 36.5 °C, (C) transmission electron micrograph at 25 °C (10 000×), (D) transmission electron micrograph at 36.5 °C (10 000×).

The particle sizes and TEM images of DRTN at 25 and 36.5 °C are shown in Figure 2. The DRTN gave the nanoparticle size of 190.0 ± 3.2 nm at 25 °C (Figure 2A). In dynamic light scattering, its particle size at 36.5 °C was not significantly different (205.7 ± 4.9 nm) from that at 25 °C (Figure 2B). From transmission electron micrographs, at 25 °C, the SLN in the DRTN exhibited the clean and clear spherical particle with a perfect border (Figure 2C). Our results suggested that the lipophilic solid SLN was surrounded by the hydrophilic poloxamer aqueous solution due to interfacial phenomenon, leading to the production of the DRTN system. Its excellent stability might be contributed by adequate amount of surfactants, including poloxamer, Tween 80 and Span 80 (Din et al., 2015a). However, at 36.5 °C, it became foggy and round-shaped particle with slightly larger size compared to that at 25 °C, suggesting that it was converted from solid to liquid form (Figure 2D). Such slight increase in particle size at 36.5 °C was due to a possible expansion of the melting SLN (Urbán-Morlán et al., 2010).

The strong gel strength and speedy gelation time are two important gel properties in the DRTN for preventing any leakage from the anus and controlling the dissolution of the drug. The gel strength of DRTN denoted its steady-state viscosity at 36.5 °C. In this study, the DRTN gave the gel strength of almost 11 000 mPa s, suggesting that it formed a strong gel at 36.5 °C. In a preliminary study, the threshold of gel strength was determined by injecting it into the rat anus at a 45° slope via a sonde fitted on a syringe, and checked for any leakage during 30 min (Seo et al., 2013). This threshold was defined as a minimum gel strength at which none leaked out from the anus for 30 min after administration, resulting from a viscosity of approximately 4000 mPa s (Yeo et al., 2013). Therefore, the gelation time, which was defined as the time required to convert from the flowing liquid to viscous gel, was determined as a time needed to attain 4000 mPa s of viscosity at 36.5 °C. In this study, the gelation time of the DRTN was about 4.8 min (raw data not shown), indicating its speedy gelation (Din et al., 2015b).

**Figure 3.**
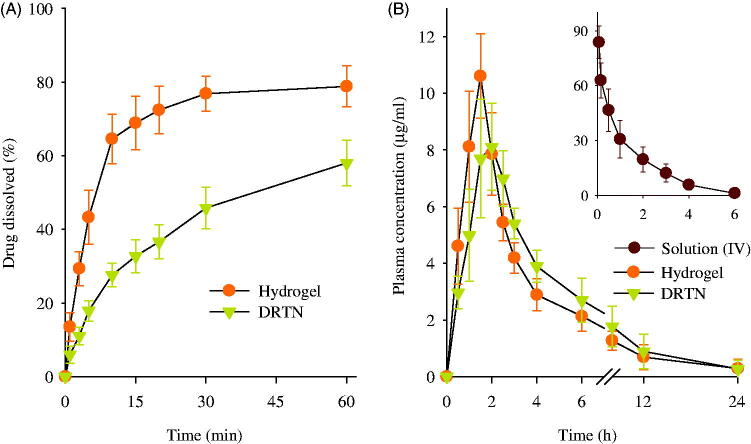
Dissolution of drug from DRTN and hydrogel (A), and plasma concentration–time profiles of irinotecan in rats after intravascular injection of solution, and rectal administration of DRTN and hydrogel (B). There were no significant differences in plasma concentration at all times between hydrogel and DRTN. Each value represents the mean ± S.D. (*n* = 6).

The dissolution of drug from DRTN was investigated compared to hydrogel (Figure 3A). At all times, the DRTN gave significantly lower dissolution rate of irinotecan compared to the hydrogel, and thus sustaining the release for 1 h (Cho et al., 2014). The DRTN could doubly regulate the discharge of the drug, first from the thermosensitive SLN and then from the thermosensitive hydrogel, leading to impeded drug dissolution and initial burst effect, and sustained release (Din et al., 2015a).

### *In vivo* evaluation in rats

The plasma concentration–time profiles of the drug, following intravenous administration of solution and rectal administration of hydrogel and DRTN at a dose of 25 mg/kg to the rats, are presented in Figure 3(B). The intravenously administered solution was used as a control so as to determine the absolute bioavailability of drug by the hydrogel and DRTN. As predicted, the solution was easily cleared from the bloodstream within 4–6 h of intravenous administration and displayed linear pharmacokinetics (Zhang et al., 2013; Ramasamy et al., 2014). The hydrogel and the DRTN achieved a maximum plasma concentration of approximately 10.5 μg/ml at 1.5 h and 8.0 μg/ml at 2.0 h, respectively, followed by gradual decrease in plasma concentration. Moreover, compared to the hydrogel, the DRTN gave lower initial plasma concentration and kept the sustained plasma concentrations of 5–8 μg/ml at 1–3 h (*p* > 0.05).

Their pharmacokinetic parameters are illustrated in Table 1. The DRTN gave lower *C*_max_ compared to the hydrogel (8.42 ± 0.88 versus 11.15 ± 1.03 μg/ml); however, they were not significantly different (*p* > 0.05). The DRTN provided significantly higher *T*_max_ than did the hydrogel (1.91 ± 0.20 versus 1.41 ± 0.20 h) (*p* < 0.05). The lower *C*_max_ and higher *T*_max_ in the DRTN suggested that the drug was slowly absorbed from the DRTN due to the delayed dissolution with no initial burst effect. Similarly, the AUC of DRTN was not significantly different from that of hydrogel (45.18 ± 12.67 versus 39.99 ± 7.69 μg h/ml), leading to their similar absolute bioavailability of drug (43.3 ± 12.7 versus 37.8 ± 7.9%). Even though the DRTN had relatively low *C*_max_ compared to the hydrogel, the similar AUC was observed owing to the sustained plasma concentrations for extended period of time (Kim et al., 2014).

**Table 1.** Pharmacokinetic parameters.

Parameters	Hydrogel	DRTN	Solution (IV)
AUC (μg h/ml)	39.38 ± 7.90	45.18 ± 12.67	104.29 ± 29.58
*T*_max_ (h)	1.41 ± 0.20	1.91 ± 0.20*	–
*C*_max_ (μg/ml)	11.15 ± 1.03	8.42 ± 0.88	83.68 ± 8.86
*t*_½_ (h)	4.16 ± 2.41	4. 61 ± 2.06	0.92 ± 0.28
*K*_el_ (h^−1^)	0.19 ± 0.09	0.18 ± 0.09	0.79 ± 0.20
Absolute bioavailability (%)	37.8 ± 7.9	43.3 ± 12.7	–

Each value represents the mean ± S.D. (*n* = 6).

AUC, area under the blood concentration–time curve; *T*_max_, time to reach the maximum plasma concentration; *C*_max_, maximum plasma concentration; *t*_1/2_, half-life; *K*_el_, elimination rate constant.

* *p* < 0.05 as compared with hydrogel. Except *T*_max_, there were no significant differences in pharmacokinetic parameters between hydrogel and DRTN.

**Figure 4.**
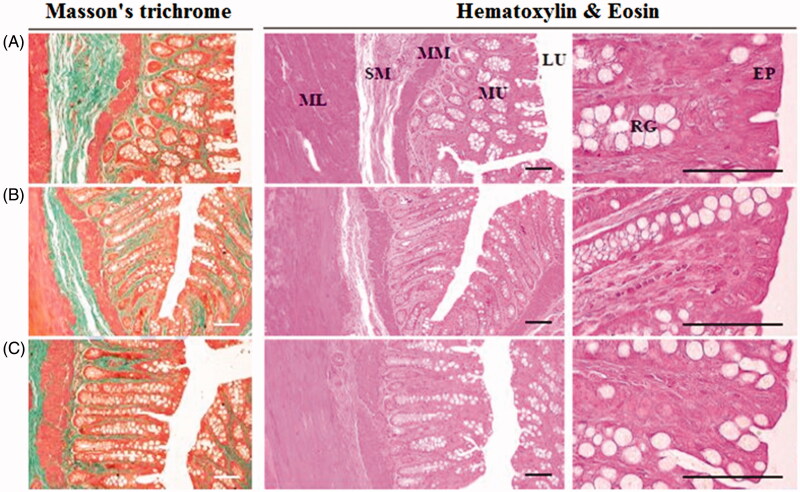
Morphology of the rat rectums: (A) control, (B) DRTN, (C) hydrogel. A simple microscope was used to investigate the rectum partitions with special emphasis on epithelium (EP), lumen (LU), rectal gland (RG), submucosa (SB), muscularis mucosa (MM) and mucosal layer (ML). Ruler bars = 120 μm.

The morphology of the DRTN and hydrogel formulation applied to the rectal mucosa of rats was compared to that of untreated (control) rat tissues (Figure 4). In this study, the morphology of solution treated rectal mucosa was not investigated, since it was intravenously administered to rats. The DRTN and hydrogel caused no abnormal damages or irritation in the rectal tissues as compared with the control (Table 2). Furthermore, no significant or considerable variations on the amount of mono nuclear cells in the lamina propria, average mucosa and epithelial thicknesses, and average collagen inhabited areas in the mucosa were observed in these formulations as related with control. Thus, the DRTN as well as the hydrogel did not induce any injury or irritation (inflammation) to the rectal mucosa. In general, the cytotoxic drug irinotecan leads to severe local injury or irritation to the exposed body tissues (Vrdoljak et al., 2009; Gao et al., 2014). Therefore, the drug in the hydrogel might induce these toxicities, since the drug in the hydrogel can be in direct contact with body tissues. However, in this study, the hydrogel could not induce such toxicities as in the case of DRTN, because the rat rectum might contain relatively wide spaces enough to avoid their frequent direct contact (Yun et al., 1999; Yong et al., 2001; Chen et al., 2012). Our results suggested that the toxic irinotecan encapsulated in the DRTN system may further avoid a direct contact with the rectal tissues, leading to no damage or irritation.

**Table 2.** Morphological analysis.

Morphology	Control	Hydrogel	DRTN
Mucosa thickness (μm)	287.8 ± 30.4	281.1 ± 26.0	289.7 ± 25.2
Epithelial thickness (μm)	37.7 ± 6.6	38.2 ± 3.9	38.0 ± 3.6
Collagen percentage (%/mm^2^)	139.3 ± 64.8	151.7 ± 82.6	135.0 ± 64.3
Mononuclear cell numbers (Cells/mm^2^)	38.1 ± 5.2	37.2 ± 5.4	37.7 ± 5.2

Each value represents the mean ± S.D. (*n* = 9).

### *In vivo* evaluation in tumor xenograft athymic nude mice

**Figure 5.**
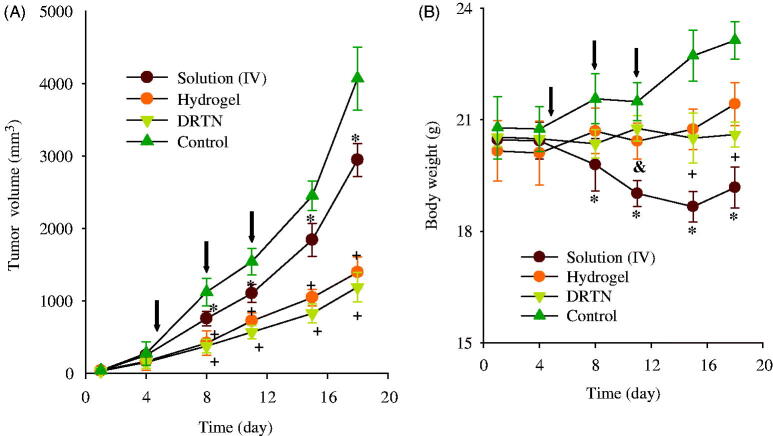
Antitumor efficacy in rats: (A) tumor volume, (B) body weight change. *p < 0.05 and +*p* < 0.05 as compared to control and solution, respectively. The arrows indicated the administration days (5, 8 and 11 days). Each value represents the mean ± S.D. (*n* = 6).

The anticancer effect of DRTN was compared to that of hydrogel and irinotecan solution in the tumor-bearing mice, respectively (Choi et al., 2015; Manchun et al., 2015). In this study, athymic nude mice were used, because these animals have been used widely for decades in the development of xenograft tumor models (Richmond & Su, 2008). Figure 5 shows the tumor volume and body weight analysis after intravenous injection of the solution, and rectal administration of DRTN and hydrogel to tumor xenograft nude mice. The tumor volume was similar (100–150 mm^3^) in all groups until they received the first dose of formulations (Figure 5A). The tumor volume rapidly increased in the control (untreated) group; however, significantly decreased (*p* < 0.05, 8–18 days) in the solution, DRTN and hydrogel-treated groups. Moreover, a significant decrease (*p* < 0.05, 8–18 days) in tumor volumes of the DRTN and hydrogel groups appeared compared to the solution-treated group. The DRTN further reduced tumor volumes as compared to the hydrogel, even though the former was not significantly different from the later. Hence, the DRTN as well as the hydrogel improved the anticancer activity of irinotecan compared to the solution.

As given in Figure 5(B), the body weight in the control group significantly increased as compared to the other groups. Additionally, the solution group led to a significant body weight loss compared to the DRTN and hydrogel solution (*p* < 0.05, 11–18 days), resulting from the toxic effects of the anticancer drug (Cetinkaya et al., 2013). However, the mice administered with DRTN and hydrogel maintained their body weights without any significant change, suggesting the potential amelioration of the toxic effects of irinotecan.

**Figure 6.**
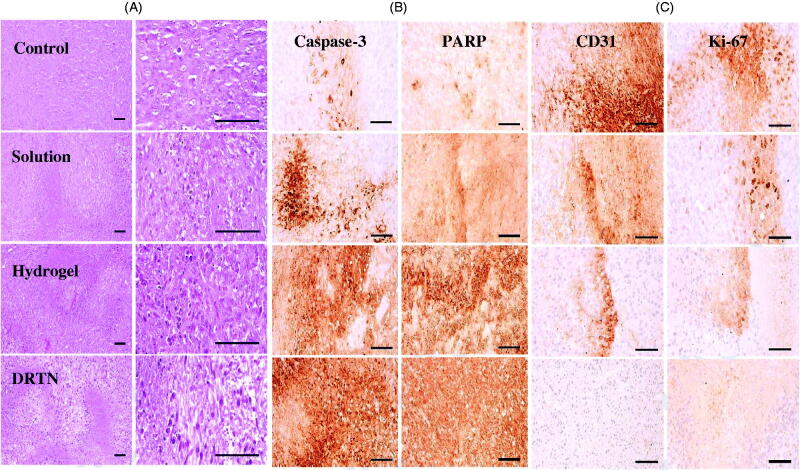
Immunohistopathology: (A) representative tumor mass histopathological changes, (B) immunoreactivities of caspase-3 and PARP-immunolabelled cells, (C) CD31 and Ki-67-immunoreactive cells. Scale bars = 120 μm.

**Figure 7.**
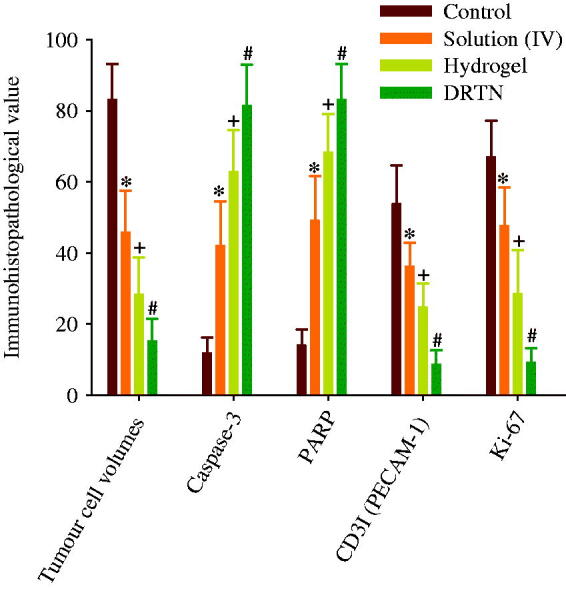
Immunohistopathological and immunohistochemical analysis. Each value represents the mean ± S.D. (*n* = 6). *p < 0.05 as compared to control. &*p* < 0.05 as compared to solution. +*p* < 0.05 as compared to control and solution. #*p* < 0.05 as compared to control, solution and hydrogel.

Our results of immunohistopathological analysis are presented in Figures 6 and 7. The untreated tumor-bearing mice were used as control. Significantly diminished tumor cell volumes, CD31- and Ki-67-positive cells (*p* < 0.05), and considerably risen caspase-3 and PARP-immunoreactive cells were demonstrated after treatment of all three types of test materials, in order of DRTN > hydrogel > solution (IV), as compared with the control (Figures 6A and 7). The decrease in the tumor volume indicated the efficiency of the developed antitumor formulation (Hua et al., 2015; Shu et al., 2015). The DRTN provided higher caspase-3 and PARP immunoreactivities than did the hydrogel as well as the solution (Figures 6B and 7) (Tran et al., 2015; Thapa et al., 2016). Additionally, the CD31 and Ki-67 were about one thirdfold diminished by the DRTN when compared to the hydrogel (Figures 6C and 7). Anti-tumor efficacy in mice presented similar reductions in the tumor volumes when treated with either DRTN or the conventional hydrogel. However, from our detailed histopathological analysis of xenograft tumors, the DRTN at the rectal administration significantly improved the anti-tumor activity of irinotecan than did the conventional hydrogel and solution owing to its sustained plasma concentrations for a long time resulting from sustained dissolution of drug without initial burst effect.

## Conclusion

The DRTN was easily administered to the rectum. Compared to the conventional hydrogel and intravenously administered solution, the DRTN achieved less of a burst effect, resulting in an enhanced anticancer efficacy with no severe side effects. Thus, this irinotecan-encapsulated DRTN would be strongly recommended as a rectal pharmaceutical product alternative to commercial intravenous injection in the treatment of rectum and colon cancer.
